# Brief interventions for smoking or alcohol moderated by history of mental health condition: a national survey of adults in Great Britain 2020–2023

**DOI:** 10.1136/bmjment-2025-301684

**Published:** 2025-07-31

**Authors:** Vera Helen Buss, Lion Shahab, Sharon Cox, Jamie Brown, Leonie S Brose

**Affiliations:** 1 Department of Behavioural Science and Health, University College London, London, UK; 2 SPECTRUM Research Consortium, Edinburgh, UK; 3 Department of Addictions, King’s College London, London, UK

**Keywords:** Cross-Sectional Studies, Substance misuse, Depression & mood disorders

## Abstract

**Background:**

Individuals with mental health conditions can experience lower life expectancy, partly due to risk factors, such as smoking and alcohol use.

**Objective:**

To assess potential differences in receiving support for smoking cessation or alcohol reduction in British general practice based on history of a mental health condition.

**Methods:**

Self-reported data were collected between October 2020 and June 2023 from the monthly cross-sectional Smoking and Alcohol Toolkit Study. The sample included 23 790 adults who smoked in the past year and/or drank at risky levels (ie, Alcohol Use Disorders Identification Test—Consumption≥5). Outcomes included the receipt of brief interventions, the recommendations provided during brief interventions and quit or cut-down attempts triggered by healthcare professionals. Logistic regression models measured associations between outcomes and lifetime mental health history, without and with adjustment for demographic and behavioural factors.

**Findings:**

Overall, 36.6% had a history of a mental health condition. About two-thirds of people with a history of a mental health condition and half of those without saw their general practitioner (GP) in the past year. Among those with a history of a mental health condition who saw their GP, 41.2% who smoked in the past year received smoking brief interventions and 7.0% who drank at risky levels received alcohol brief interventions. Receipt of smoking brief interventions was similar by history of mental health condition (with 41.2% vs without 41.1%). Individuals with a history of a mental health condition compared with those without had higher odds of receiving alcohol brief interventions (7.0% vs 2.8%, adjusted OR=2.69, 95% CI: 2.17 to 3.34) and receiving more comprehensive support as part of the intervention.

**Discussion:**

Among respondents with a history of a mental health condition, only around 4 in 10 smokers who visited their GP received brief interventions from their GP and 1 in 20 for alcohol.

**Clinical implications:**

Considering the links between smoking or risky drinking and mental health conditions, healthcare professionals should increase screening and brief advice to reduce health disparities.

**WHAT IS ALREADY KNOWN ON THIS TOPIC**

- Helping people with mental health conditions to quit smoking or reduce alcohol consumption can improve both physical and mental health. General practitioners are crucial in screening and providing brief interventions, although evidence suggests that rates of advice given are low.

**WHAT THIS STUDY ADDS**

- While more people who smoked received brief interventions than those who drank at risk levels, individuals with a history of mental health conditions were more likely to receive alcohol brief interventions and the support received was more intensive, while rates for smoking brief interventions were similar across groups.

**HOW THIS STUDY MIGHT AFFECT RESEARCH, PRACTICE OR POLICY**

- The study underscores the need for increasing screening and providing brief interventions for people with mental health conditions who smoke or drink at risky levels, addressing a missed opportunity to reduce health disparities.

## Introduction

 People with mental health conditions, especially those with severe ones, have a substantially lower life expectancy than those without.^1^ Cigarette smoking and alcohol use are key contributors to this difference.^2^ Providing support to people with mental health conditions to quit smoking or cut down their alcohol consumption can help to reduce health disparities. One important point of contact is general practitioners (GPs). The UK National Institute for Health and Care Excellence guidelines recommend that GPs should screen their patients regarding smoking and alcohol consumption and provide brief advice.^3 4^

For smoking cessation in primary care, the UK guidelines recommend discussing different intervention options (behavioural support, medicinally licenced products (cytisine, nicotine replacement therapy, varenicline, bupropion) or nicotine-containing e-cigarettes) or referring to a free local stop-smoking support service.^3^ Generally, GPs can prescribe any of the medicinally licensed products,^3^ which will either be free of charge or require the patients to pay a standard prescription charge (currently £9.90).^5^ These guidelines apply across all of Great Britain.

For alcohol brief intervention, the UK guidelines recommend assessing alcohol consumption using the Alcohol Use Disorders Identification Test—Consumption (AUDIT-C) Score, informing patients about their related health risk, and if they drink above lower risk levels, providing them with an information leaflet about the benefits of cutting down.^4 6 7^ However, if patients are potentially alcohol dependent, they should be referred to specialist alcohol assessment. Further, patients experiencing physical or psychological harm as a result of drinking alcohol should be referred to specialist services.^4^ Guidelines stress the importance of considering the risk of stigma and discrimination associated with alcohol use disorders and ensuring that confidentiality, privacy and dignity are maintained.^7^ According to a study, most people in England are aware that UK low-risk drinking guidelines exist, but they do not know what the recommended weekly limit is.^8^

Cross-sectional data from 2020 to 2022 showed 29% of adults living in England had been diagnosed with a mental health condition in their lifetime.^9^ Among people who drank at risky levels (operationalised as AUDIT-C≥5^7 10^), 34% had a diagnosed mental health condition and among those who smoked, the figure was 42%.^9^ Helping people with mental health conditions to stop smoking or reduce their drinking can bring improvements to their physical health, and two systematic reviews have shown that quitting smoking can also improve people’s mental well-being, even for those with severe mental illness.^11 12^ Therefore, mental health and smoking or drinking could be treated in parallel. A study based on British general practice patient data from 2009 to 2010 found that half the patients with a mental health diagnosis who smoked had a record of cessation advice compared with only one-third of those without such a diagnosis.^13^

Among adults who smoked in England in 2012, those who had depression or anxiety had higher odds of reporting being referred to stop smoking services by their GP than those without depression and anxiety, but there was no difference between the groups in receiving general advice without additional support, being advised to talk to a practice nurse or being advised to take nicotine replacement therapy.^14^ In a more recent study based on data from 2020 to 2022, people living in England with a history of a mental health condition had higher odds of receiving brief advice from their GP for smoking among those who smoked in the past year and possibly for alcohol among those who drank at risky levels than those without a mental health condition.^15^ In a cohort study using English primary care records from 2007 to 2014 found that among patients who smoked, approximately 2–3% had received a referral to stop smoking services and 10–12% had received a prescription for stop smoking medication, without noteworthy differences between those with a history of a severe mental illness, those with depression or those with neither.^16^ Overall, there appears to be a scarcity of published data on alcohol brief interventions by mental health status in Great Britain.

Beyond frequency, the type of advice received is also important. Besides GPs, other healthcare professionals are also advised to provide brief interventions, such as mental health and acute care providers.^6^ To be effective, brief interventions by health professionals need to trigger attempts to quit smoking or reduce alcohol consumption. Therefore, this study aimed to assess in Great Britain between 2020 and 2023:

1. Among those who visited their GP in the past year, associations between receiving (a) a smoking or (b) alcohol brief intervention and having a history of a mental health condition.
2. Among those who received a brief intervention by their GP in the past year, associations between the different types of recommendation received by their GP during the brief intervention and history of a mental health condition.
3. Among those who tried to quit smoking or reduce their alcohol consumption in the past year, associations between the most recent attempt to (a) stop smoking or (b) restrict alcohol use triggered by a healthcare professional and a history of mental health condition.

## Methods

### Study design

Data for this study were collected between October 2020 and June 2023 as part of the Smoking and Alcohol Toolkit Study, an ongoing monthly cross-sectional population-based survey. The survey captures a range of data on smoking-related and drinking-related behaviours from a new sample of adults each month. During the period included in the study, participants were also asked about their history of a mental health diagnosis. The sampling strategy consists of a combination of random location and quota sampling. A market research company conducted the interviews with survey participants via telephone as part of their telephone omnibus survey. Each month, a new representative sample is recruited. The research team received anonymised data. More details on the survey methods are published elsewhere.^17^ The manuscript followed the STrengthening the Reporting of OBservational studies in Epidemiology statement.^18^ The study protocol, including the analysis plan, was preregistered and is publicly available together with the syntax and data on the Open Science Framework.^19^

### Outcome variables and covariates

All variables were self-reported. Participants were included if they smoked in the past year or if they drank at risky levels (AUDIT-C Score≥5^7 10^). Details on the wording of the questions and classification are presented in online supplemental file 1. The outcome measures were: visiting a GP in the past year, receipt of GP-based brief interventions for smoking or drinking, type of support received and most recent attempt to stop smoking/reduce drinking triggered by a healthcare professional (see table 1).

**Table 1:** Outcomes, the survey questions with which they were measured and how they were classified

Outcome	Survey question	Classification
Visiting GP in past year (past-year smoking)	“Has your GP spoken to you about smoking in the past year (ie, last 12 months)? Yes, he/she suggested that I go to a specialist stop smoking advisor or group. Yes, he/she suggested that I see a nurse in the practice. Yes, he/she offered me a prescription for Champix, Zyban, a nicotine patch, nicotine gum or another nicotine product. Yes, he/she suggested that I use an e-cigarette. Yes, he/she advised me to stop but did not offer anything. Yes, he/she asked me about my smoking but did not advise me to stop smoking. No, I have seen my GP in the last year but he/she has not spoken to me about smoking. No, I have not seen my GP in the last year. Don’t know.”	Those who responded (i)–(vii) were classified as having visited their GP in the past year. Those who replied (viii) were categorised as having not visited their GP.
Receipt of GP-based brief intervention (for past-year smoking)		Participants who visited their GP were classified as having received a smoking brief intervention if they selected one of answer options (i)–(v). If they selected (vi) or (vii), they were classified as not having received a smoking brief intervention.
Type of support received during brief intervention (for past-year smoking)		Type of support to quit smoking received as part of the brief intervention among those who visited their GP was categorised into ‘advice only’ (option (v)), ‘e-cigarette’ (option (iv)), ‘prescription’ (option (iii)), ‘referral to practice nurse’ (option (ii)) or ‘referral to stop smoking service’ (option (i)).
Most recent quit attempt triggered by healthcare professional (for past-year smoking)	“How many serious attempts to stop smoking have you made in the last 12 months? By serious attempt I mean you decided that you would try to make sure you never smoked again. Please include any attempt that you are currently making and please include any successful attempt made within the last year.” If participants said at least one, they were classified as having made at least one serious attempt in the past 12 months. They were then asked, “Which of the following do you think contributed to you making the most recent quit attempt?” and provided with a list of options (multiple answer options possible).	The most recent attempt was triggered by a healthcare professional if the response was “Advice from a GP\health professional”; otherwise, not.
Visiting GP in the past year (for risky drinking)	“In the last 12 months, has a doctor or other health worker within your GP surgery discussed your drinking? No Yes, a doctor or other health worker within my GP surgery asked about my drinking. Yes, a doctor or other health worker within my GP surgery offered advice about cutting down on my drinking. Yes, a doctor or other health worker within my GP surgery offered help or support within the surgery to help me cut down. Yes, a doctor or other health worker within my GP surgery referred me to an alcohol service or advised me to seek specialist help. Don’t know Refused” If someone answered “No”, they were asked “You said a doctor or other health worker within your GP surgery has not discussed your drinking with you in the last 12 months. Please could you confirm which of the following statements applies to you: I have not seen a doctor or health worker within my GP surgery in the last 12 months. I have seen a doctor or health worker within my GP surgery in the last 12 months but did not discuss my drinking. Don’t know”	Participants were classified as having visited their GP in the past year if they answered one of options (ii)–(iv) to the first question or (ii) to the follow-up question. They were classified as not having visited their GP if they replied (i) to the follow-up question.
Receipt of GP-based brief intervention (for risky drinking)		Those who visited their GP were classified as having received an alcohol brief intervention if they selected one of answer options (iii)–(v). If they only selected (i) to the first question and (ii) to the follow-up question or (ii) to the first question, they were classified as not having received an alcohol brief intervention.
Type of support received during brief intervention (for risky drinking)		Type of support to reduce consumption received as part of the brief intervention among those who visited their GP was categorised into ‘advice only’ (option (iii)), ‘help or support within the practice’ (option (iv)) or ‘referral to alcohol service/specialist’ (option (v)).
Most recent quit attempt triggered by healthcare professional (for risky drinking)	(1) “Are you currently trying to restrict your alcohol consumption for example, by drinking less, choosing lower strength alcohol or using smaller glasses?”; and (2) “How many attempts to restrict your alcohol consumption have you made in the last 12 months (eg, by drinking less, choosing lower strength alcohol or using smaller glasses)? Please include all attempts you have made in the last 12 months, whether or not they were successful, AND any attempt that you are currently making.” If participants said yes to (2) or at least one to (2), they were classified as having made an attempt in the past 12 months. They were then asked, “Which of the following, if any, do you think contributed to you making the most recent attempt to restrict your alcohol consumption?”, and provided with a list of answer options (multiple answer options possible).	The most recent attempt was triggered by a healthcare professional if the response was “Advice from a doctor\health worker”; otherwise, not.

GP, general practitioner; .

People were classified as having a history of a mental health condition if they reported at least one condition to the question “Since the age of 16, which of the following, if any, has a doctor or health professional ever told you that you had?

1. depression
2. anxiety
3. obsessive compulsive disorder
4. panic disorder or a phobia
5. post-traumatic stress disorder
6. psychosis
7. personality disorder
8. attention deficit hyperactivity disorder
9. an eating disorder
10. alcohol misuse or dependence
11. drug use or dependence
12. problem gambling
13. autism or autism spectrum disorder
14. bipolar disorder (previously known as manic depression)”.

The question was adapted from other surveys.^20 21^ The list of answer options is based on a report on adult psychiatric morbidity in England and in line with ICD-10 recognised mental and behavioural disorders.^21^ If none of the answer options were selected, participants were classified as not having a history of a diagnosed mental health condition. Among people classified as risky drinking, those who only selected answer option 10 (‘alcohol misuse or dependence’) but none of the other answer options were not counted separately as having a history of a mental health condition (n=296). In a sensitivity analysis, we separately conducted the analysis for people who reported having a history of depression or anxiety (option (i) or (ii)).

Other variables used in the analysis included age, gender, socioeconomic position, nation, tobacco dependence, alcohol consumption level and survey wave. We modelled age and survey wave using restricted cubic splines with three knots (placed at the minimum, median and maximum for age; and the beginning, middle and end for time). More information is provided in the online supplemental file 1.

### Analysis

The analyses were conducted in RStudio (version 2022.07.1, R V.4.2.1). Responses noted as “Don’t know” or “Refused” were considered missing (see online supplemental table S1 and figures S1–S3 for missing values and patterns of missingness). In five waves, only data from Scotland and Wales were available, because at these time points (ie, May, July, September, November and December 2022), outcome measures related to alcohol were not assessed in England. We imputed missing data using multivariate imputation by chained equations by creating five imputed data sets using the mice package (details in online supplemental table S2). Each imputed data set was analysed separately, and estimates combined using Rubin’s rules. We assumed data on the history of a mental health condition were missing not at random; assuming among people with missing data, the odds of having a history of a mental health condition were 1.5 times higher than what the model otherwise predicted. This assumption was based on findings by Perales Perez and Baffour.^22^ As sensitivity analyses, we (1) conducted a complete case analysis and (2) imputed data assuming all values were missing at random. We applied raking, a weighting technique using iterative proportional fitting, setting separate representative target profiles for Great Britain (based on gender, working status, prevalence of children in the household, age, social grade and region) and repeating the process until all variables meet the specified targets. Unweighted estimates are presented in online supplemental tables S3 and S4.

#### GP visit and receipt of brief intervention (RQ1)

We assessed the proportion of participants who reported having visited their GP in the past 12 months among (1) those who smoked in the past year and (2) those who drank at risky levels, stratified by history of mental health condition. Among those who visited their GP, we assessed the proportion who reported (3) receiving a smoking brief intervention among those who smoked in the past year and (4) who reported receiving an alcohol brief intervention among those drinking at risky levels, stratified by history of mental health condition. We estimated the unadjusted and adjusted (for age, gender, social grade, nation, survey wave and tobacco dependence (for smoking brief intervention) or alcohol consumption level (for alcohol brief intervention)) association between receiving a brief intervention and history of mental health condition, separately for those who smoked in the past year and those who drank at risky levels, using logistic regression. In a sensitivity analysis, we also adjusted for risky drinking when assessing receipt of smoking brief interventions and past-year smoking when assessing receipt of alcohol brief interventions.

In addition to the prespecified analysis, we also estimated the unadjusted and adjusted association between receiving a brief intervention and history of mental health condition among people who smoked in the past year and drank at risky levels.

#### Support received (RQ2)

Among everyone who received a smoking or alcohol brief intervention, we assessed the proportion who reported having received each type of support during the brief intervention by their GP, stratified by history of mental health condition. We estimated the unadjusted and adjusted associations between each type of support received and history of mental health condition using logistic regression.

#### Quit/restrict attempt triggered by healthcare professional (RQ3)

We assessed the proportion of participants (1) who tried to stop smoking in the past 12 months among those who smoked in the past year and (2) who tried to restrict their drinking in the past 12 months among those who drank at risky levels, stratified by history of mental health condition. Among those who tried to stop or restrict their consumption, we assessed the proportion of people whose attempt was triggered by a healthcare professional (ie, any healthcare professional, including but not limited to GPs), separately for smoking and drinking and stratified by history of mental health condition. We estimated the unadjusted and adjusted association between attempts triggered by healthcare professionals and history of mental health condition, separately for those who smoked in the past year and those who drank at risky levels, using logistic regression.

## Results

We included 23 790 participants (unweighted) who smoked in the past year (44.3% of the sample, 95% CI: 43.6% to 45.0%) or drank at increasing-or-higher-risk levels (74.8% of the sample, 95% CI: 74.2% to 75.4%). Overall, 11 276 (36.6%, 95% CI: 36.0% to 37.3%) participants had a history of a mental health condition. Table 2 shows the weighted sociodemographic characteristics of participants.

**Table 2:** Characteristics of adults who smoked or drank at increasing-or-higher-risk levels depending on history of a mental health condition (N_unweighted_=23 790, data in table weighted)

	Only past-year smoking, % (95% CI)		Only risky drinking, % (95% CI)		Past-year smoking and risky drinking, % (95% CI)	
	History of mental health condition					
	Yes (n=2953)	No (n=3079)	Yes (n=3738)	No (n=9578)	Yes (n=2074)	No (n=2503)
18–24 years	15.4 (13.9 to 16.9)	11.6 (10.2 to 13.0)	12.9 (11.7 to 14.0)	9.2 (8.6 to 9.9)	25.5 (23.4 to 27.7)	17.0 (15.3 to 18.7)
25–34 years	27.8 (25.9 to 29.7)	21.5 (19.8 to 23.3)	18.8 (17.4 to 20.2)	14.3 (13.5 to 15.1)	28.7 (26.5 to 31.0)	25.0 (23.0 to 27.0)
35–44 years	19.0 (17.4 to 20.7)	15.7 (14.2 to 17.2)	19.1 (17.7 to 20.5)	15.8 (15.0 to 16.6)	18.8 (16.9 to 20.7)	18.6 (16.9 to 20.4)
45–54 years	15.8 (14.4 to 17.3)	14.6 (13.3 to 16.0)	19.5 (18.2 to 20.9)	20.8 (19.9 to 21.6)	14.6 (13.0 to 16.3)	17.2 (15.6 to 18.8)
55–64 years	12.1 (10.9 to 13.3)	14.1 (12.8 to 15.4)	16.8 (15.6 to 18.0)	19.1 (18.3 to 19.9)	8.2 (6.9 to 9.5)	13.1 (11.7 to 14.5)
65+ years	9.9 (8.8 to 11.0)	22.5 (20.9 to 24.0)	12.9 (11.8 to 14.0)	20.8 (20.0 to 21.6)	4.1 (3.2 to 5.0)	9.0 (7.9 to 10.1)
Women	57.8 (55.8 to 59.9)	45.5 (43.5 to 47.4)	51.0 (49.3 to 52.8)	32.9 (32.0 to 33.9)	46.1 (43.7 to 48.5)	31.2 (29.2 to 33.2)
Men	40.7 (38.6 to 42.7)	54.2 (52.2 to 56.2)	48.0 (46.3 to 49.7)	66.7 (65.7 to 67.7)	51.1 (48.7 to 53.5)	68.3 (66.3 to 70.3)
Non-binary	1.5 (1.1 to 2.0)	0.3 (0.1 to 0.5)	1.0 (0.7 to 1.3)	0.3 (0.2 to 0.5)	2.8 (2.1 to 3.5)	0.5 (0.2 to 0.8)
ABC1	35.4 (33.6 to 37.2)	40.9 (39.0 to 42.8)	61.8 (60.1 to 63.6)	66.4 (65.4 to 67.5)	47.1 (44.7 to 49.5)	52.8 (50.6 to 55.0)
C2DE	64.6 (62.8 to 66.4)	59.1 (57.2 to 61.0)	38.2 (36.4 to 39.9)	33.6 (32.5 to 34.6)	52.9 (50.5 to 55.3)	47.2 (45.0 to 49.4)
England	85.0 (83.7 to 86.3)	85.3 (84.0 to 86.5)	82.3 (81.1 to 83.5)	82.4 (81.7 to 83.1)	85.4 (83.8 to 86.9)	84.9 (83.5 to 86.3)
Scotland	9.2 (8.2 to 10.3)	8.9 (8.0 to 9.9)	11.9 (10.9 to 12.9)	12.1 (11.5 to 12.7)	9.0 (7.8 to 10.2)	10.1 (8.9 to 11.3)
Wales	5.8 (4.9 to 6.6)	5.8 (4.9 to 6.6)	5.8 (5.0 to 6.5)	5.5 (5.1 to 6.0)	5.6 (4.6 to 6.7)	5.0 (4.1 to 5.9)

Social grades ABC1: more socioeconomically advantaged. Social grades C2DE: less socioeconomically advantaged.

### GP visit and receipt of brief intervention (RQ1)

Two-thirds of people with a mental health condition visited their GP in the past year and about half of those without a mental health condition (table 3). Among those who visited their GP and smoked in the past year, 4 out of 10 received a smoking brief intervention, and the rate was similar between those with and without mental health conditions. Among those who visited their GP and drank at risky levels, people with a mental health condition were more likely to receive an alcohol brief intervention (7.0%, 95% CI: 6.1% to 7.9%) than those without (2.8%, 95% CI: 2.4% to 3.2%; adjusted OR (OR_adj_)=2.69, 95% CI: 2.17 to 3.34).

**Table 3:** GP visit and receipt of brief intervention by history of mental health condition for people who smoked in the past year (n_weighted_=9936) or who drink at risky levels (n_weighted_=18 146)

	History of mental health condition			
	Yes, % (95% CI)	No, % (95% CI)	OR (95% CI), ref: no	OR_adj_* (95% CI), ref: no
Past-year smoking				
GP visit	65.2 (63.7 to 66.7)	50.3 (48.8 to 51.8)	‒	‒
SBI receipt	41.2 (39.3 to 43.1)	41.1 (39.0 to 43.1)	1.00 (0.89 to 1.13)	1.02 (0.90 to 1.15)
Risky drinking				
GP visit	69.5 (68.2 to 70.8)	55.1 (54.1 to 56.0)	‒	‒
ABI receipt	7.0 (6.1 to 7.9)	2.8 (2.4 to 3.2)	2.59 (2.11 to 3.19)	2.69 (2.17 to 3.34)

* Adjusted for age, gender, social grade, tobacco dependence or Alcohol Use Disorders Identification Test—Consumption Score, nation and survey wave.

ABI, alcohol brief intervention; GP, general practitioner; OR_adj_, adjusted OR; SBI, smoking brief intervention.

### Support received (RQ2)

The type of support GPs provided to their patients during a smoking brief intervention was similar by history of a mental health condition (table 4). In both groups, most commonly people either received advice without additional support or a referral to stop smoking services (about 40% each). Further, about 1 in 4 received a prescription for stop smoking medication, 1 in 5 received a referral to a practice nurse and 1 in 10 received a recommendation to use an e-cigarette.

**Table 4:** Type of support suggested by GP during smoking brief interventions (n_weighted_=2504) or alcohol brief intervention (n_weighted_=469), by history of mental health condition

	History of mental health condition			
	Yes, % (95% CI)	No, % (95% CI)	OR (95% CI), ref: no	OR_adj_* (95% CI), ref: no
SBI				
Advice only	41.3 (38.4 to 44.3)	41.0 (37.8 to 44.2)	1.01 (0.85 to 1.21)	1.01 (0.84 to 1.23)
E-cigarette	11.0 (9.2 to 12.9)	8.1 (6.3 to 9.9)	1.40 (1.03 to 1.91)	1.30 (0.93 to 1.82)
Prescription	24.3 (21.7 to 26.8)	23.3 (20.6 to 26.0)	1.05 (0.86 to 1.30)	1.10 (0.88 to 1.38)
Referral to practice nurse	18.5 (16.1 to 20.8)	18.8 (16.3 to 21.3)	0.98 (0.78 to 1.22)	1.04 (0.82 to 1.32)
Referral to SSS	38.5 (35.6 to 41.4)	36.2 (33.1 to 39.4)	1.10 (0.92 to 1.32)	1.05 (0.86 to 1.27)
ABI				
Advice only	69.3 (63.2 to 75.4)	90.1 (85.4 to 94.8)	0.25 (0.14 to 0.45)	0.31 (0.16 to 0.60)
Support within the practice	31.6 (25.6 to 37.7)	11.3 (6.3 to 16.2)	3.65 (2.07 to 6.44)	3.59 (1.89 to 6.80)
Referral to alcohol service/ specialist	35.7 (29.4 to 42.1)	6.0 (2.1 to 9.9)	8.74 (4.17 to 18.31)	8.85 (4.00 to 19.58)

* Adjusted for age, gender, social grade, tobacco dependence or Alcohol Use Disorders Identification Test—Consumption Score, nation and survey wave.

ABI, alcohol brief intervention; GP, general practitioner; OR_adj_, adjusted OR; SBI, smoking brief intervention; SSS, stop smoking service.

The support GPs provided to their patients during alcohol brief interventions differed depending on the patients’ history of a mental health condition, but, overall, most of both groups received advice only (table 4). Those with a history of a mental condition had higher odds of receiving help or support within the practice (OR_adj_=3.59, 95% CI: 1.89 to 6.80) and a referral to an alcohol service or specialist (OR_adj_=8.85, 95% CI: 4.00 to 19.58). In comparison, people with a mental health condition had lower odds of receiving only advice from their GP about their drinking (OR_adj_=0.31, 95% CI: 0.16 to 0.60).

### Quit/restrict attempt triggered by healthcare professional (RQ3)

Among people smoking in the past year, 40.2% (95% CI: 38.6% to 41.7%) with and 34.5% (95% CI: 33.1% to 35.9%) without a history of a mental health condition reported a quit attempt in the past year. Independent of whether people had a history of a mental health condition, 1 in 10 of those who tried to quit smoking in the past year stated that their attempt was triggered by a healthcare professional (with: 11.3%, 95% CI: 9.7% to 12.9% vs without: 9.6%, 95% CI: 8.1% to 11.1%; OR=1.21, 95% CI: 0.95 to 1.53; OR_adj_=1.19, 95% CI: 0.92 to 1.54).

Among those who drank at risky levels, 41.2% (95% CI: 39.8% to 42.5%) with and 31.6% (95% CI: 30.7% to 32.4%) without a history of a mental health condition tried to reduce their consumption in the past year. People with a history of a mental health condition had higher odds of reporting that their attempt was triggered by a healthcare professional (9.0%, 95% CI: 7.7% to 10.3% vs 5.4%, 95% CI: 4.7% to 6.2%; OR=1.73, 95% CI: 1.40 to 2.14; OR_adj_=1.99, 95% CI: 1.60 to 2.48).

### Sensitivity analyses

The results of the sensitivity analyses seem to be overall consistent with the results of the main analysis (see online supplemental tables S5–S13). A noteworthy difference was that in the complete case analysis (online supplemental table S8), the ORs for receiving support within the practice (OR_adj_=4.36, 95% CI: 2.14 to 8.91) or referral to alcohol service or specialist (OR_adj_=11.70, 95% CI: 4.47 to 30.80) as part of an alcohol brief intervention were somewhat larger than in the main analysis (OR_adj_=3.59, 95% CI: 1.89 to 6.80; and OR_adj_=8.85, 95% CI: 4.00 to 19.58), but the confidence intervals were overlapping and there was more uncertainty around the estimates of the complete case analysis due to the smaller sample size. There were no noteworthy differences when specifically looking at people with a history of depression or anxiety rather than any mental health condition (online supplemental table S6).

Additionally, adjusting for drinking or smoking status, respectively, did not impact the magnitude of the association between receipt of brief interventions and history of a mental health condition, suggesting that the odds of receiving a smoking or alcohol brief intervention depending on a history of a mental health condition were not significantly affected by whether someone additionally engaged in the other behaviour (online supplemental table S5). Among people who smoked in the past year and drank at risky levels, 73.5% (95% CI: 71.3% to 75.6%) with and 57.2% (95% CI: 55.1% to 59.4%) without a history of a mental health condition visited their GP in the past year (online supplemental table S11). Among these, about one-third from each group received a smoking brief intervention (with: 32.4%, 95% CI: 29.7% to 35.0%; without: 31.5%, 95% CI: 28.8% to 34.2%; OR_adj_=1.02, 95% CI: 0.84 to 1.23) which is overall a lower prevalence than among all who smoked in the past year. In contrast, both groups had a slightly higher prevalence of receiving an alcohol brief intervention than all who drank at risky levels, but the ratio was similar with those with a history of a mental health condition having higher odds (9.1%, 95% CI: 7.5% to 10.8% vs 3.4%, 95% CI: 2.3% to 4.4%; OR_adj_=2.97, 95% CI: 2.01 to 4.38).

## Discussion

The receipt of a smoking brief intervention, the type of recommendation given by the GP as part of the smoking brief intervention and the rate of smoking quit attempts triggered by healthcare professionals were similar by history of a mental health condition. Generally, about 4 out of 10 people who smoked and visited their GP in the past year received a smoking brief intervention. Most commonly, they only received advice with no additional support, or they were referred to stop smoking services.

Alcohol brief interventions were less common than smoking brief interventions. However, the odds of receiving such an intervention were more than double, and the type of support offered by GPs tended to be more intensive for those with a history of a mental health condition than for those without these conditions. While about 7% of people with a mental health condition who drank at risky levels and visited their GP in the past year reported receiving an alcohol brief intervention, only about 3% of those without a history of a mental health condition did so. Those who received an alcohol brief intervention and had a history of a mental health condition had higher odds of receiving further support within the practice or a referral to alcohol services or specialists than others who rather received advice from their GP without being referred on. People with a history of a mental health condition also had higher odds of alcohol reduction attempts being triggered by healthcare professionals.

Our results are in line with previous research showing brief interventions for smoking are more commonly offered than brief interventions for alcohol.^15 23 24^ One plausible explanation is that GPs may feel more comfortable discussing smoking with their patients than discussing alcohol consumption.^24^,^26^ Reasons for that might include: GPs being concerned that discussing drinking, more so than smoking, might offend or alienate patients; there might be more established guidelines, training resources and intervention options for smoking cessation than alcohol reduction; GPs might also have perceived smoking cessation as a greater health priority than alcohol reduction and personal views on alcohol and its role in society may influence their willingness to address it with their patients.^27^

A previous study reported higher odds for people who were ever diagnosed with a mental health condition to have received a smoking brief intervention and potentially higher odds of receiving an alcohol brief intervention.^15^ However, a key difference was that the previous finding focused on all who smoked in the past year rather than only those who visited their GP. In the current study, a higher percentage of people with a mental health history who saw their GP in the past year than people without a mental health history. It is likely that, in absolute terms, more smokers with mental health history received a brief intervention than those without.^15^ Similarly to our study, a study using data from 2012 found that there were no associations between receiving a smoking brief intervention and having anxiety or depression.^14^ While the previous study found that among those who saw their GP, people with anxiety or depression had higher odds of being advised to try a stop smoking service, we did not find such an association. However, in our study, there was some indication that they might have higher odds of getting a recommendation to use an e-cigarette, which was not widely available at the time of the previous survey and therefore not included as an answer option.

People with a history of a mental health condition having higher odds of receiving an alcohol brief intervention compared with those without might be explained by the clinicians’ awareness of the common co-occurrence of risky alcohol use and other mental health conditions.^28^ GPs also seemed more likely to provide comprehensive support for alcohol use to patients with a history of a mental health condition by referring them to further support provided within the practice or to a specialist or substance use service. Potentially, patients may also experience barriers to accessing mental health specialist services unless they have addressed their co-occurring alcohol use first, which may explain why GPs are more likely to refer patients with a mental health condition to specialist services for alcohol treatment than people without a mental health condition. It is also possible that patients with a mental health condition initiated the conversation about support for alcohol use rather than the GPs, because otherwise they would not be able to access mental health specialist services. While it was beyond the scope of this study, future research could usefully explore potential mechanisms underpinning the observed differences in receipt of brief alcohol interventions.

According to a US study using data from 2020 to 2023, there was no difference in the odds of receiving an alcohol-related assessment in primary care between people with zero or one chronic condition compared with those with multiple chronic conditions.^29^ Regarding smoking, a US primary care study using data from 2014 to 2016 found that patients with hypertension, asthma or chronic obstructive pulmonary disease, and hyperlipidaemia had higher odds of receiving smoking cessation counselling than those without condition, but there was no difference for those with diabetes, coronary artery disease, cancer, psychiatric disorder or substance use disorder compared with no condition.^30^ In a Scottish primary care study using data from 2006 to 2007, among people with coronary heart disease who smoked, those with other comorbid physical health conditions had higher odds of receiving smoking cessation advice, but those with mental health conditions had lower odds than those without.^31^

While it is positive from a health disparities point of view that people with a history of a mental health condition have higher odds of receiving alcohol brief interventions than those without, overall, the receipt rates are concerningly low, especially for alcohol but also for smoking. Given the well-established benefits of smoking cessation and alcohol reduction both on physical and mental health,^11 12^ the low intervention rates suggest missed opportunities in general practice. This study underscores the need for healthcare professionals to make every contact count, as currently only a minority of attempts to quit smoking or reduce alcohol consumption are triggered by healthcare professionals. This indicates that the problem extends beyond primary care, requiring a more comprehensive approach to support people with a mental health condition. However, it is important to recognise the current workforce challenges in an overburdened healthcare system. These include increased demand combined with limited consultation time, which can make it extremely difficult to make every contact count. Therefore, wider system issues such as inadequate funding and lack of staff are likely to contribute to low brief intervention rates.

### Limitations

All measures are self-reported and there is a risk of recall bias or under-reporting, particularly on mental health, smoking and drinking, which are all associated with stigma. For some analyses, sample sizes were small, resulting in a greater level of uncertainty around the estimates. While for smoking, people were included who quit within the past year, for alcohol, only people who still drank at risky levels at the time of the survey were included. Participants who stopped smoking in the past year could have potentially quit due to a brief intervention, but it is also possible that they had already stopped at the time of their GP visit and were therefore ineligible for a smoking brief intervention. It is also possible that people who drank at risky levels at the time of the survey consumed below the risk threshold at the time of their GP visit and were therefore ineligible for an alcohol brief intervention. The analyses included many statistical tests based on the same data, which may have resulted in some being statistically significant just by chance. The study includes data collected during the acute phase of the pandemic, which may have differentially impacted the delivery rate of brief interventions to those with and without a mental health history.

We assessed the history of a mental health condition rather than the status quo. Therefore, it is possible that participants were diagnosed with a mental health condition in the past but were no longer affected by it at the time of their GP visit. While the study considered whether people saw their GP in the past year, it did not account for the number of visits, which may have an impact on the likelihood of receiving a brief intervention within the past year.^13 30^ The main analysis included drug use or dependence and problem gambling for the classification of mental health condition, which are potentially distinct from other mental health conditions included. However, a sensitivity analysis including only depression or anxiety (the two most common mental health conditions in Great Britain) showed a similar pattern of results. There is a risk of uncontrolled confounding due to unmeasured confounders, such as physical comorbidities and the number of times a participant visited their GP in the past year. On the basis of this study, no statements can be made about the quality or effects of the brief interventions delivered by GPs, although there is an established evidence base on their effects.^3 4^

## Conclusions

Around 4 in 10 eligible smokers received a brief intervention from their GP compared with 1 in 20 for alcohol. Receiving alcohol, but not smoking, brief interventions were more likely among individuals with a history of a mental health condition. Considering the immense burden of disease caused by smoking and alcohol use and the high prevalence of co-occurrence of tobacco smoking or riskier drinking and mental health conditions, healthcare professionals should put more emphasis on screening and providing brief advice, especially among people with mental health conditions, to reduce health disparities.

## Supplementary material

10.1136/bmjment-2025-301684 online supplemental file 1

## Data Availability

Data are available in a public, open-access repository.
