# Supplementary material for: Brief interventions for smoking or alcohol moderated by history of mental health condition: a national survey of adults in Great Britain 2020–2023
Source: BMJ Ment Health. 2025 Jul 31;28(1):e301684. doi: 10.1136/bmjment-2025-301684 (PMC12314975; doi:10.1136/bmjment-2025-301684)
Supplement: online supplemental file 1 [file bmjment-28-1-s001.pdf]

Supplementary material

Article: Brief intervention for smoking or alcohol moderated by history of mental health condition: A national survey of adults in Britain 2020-2023

Table of Contents

1. Covariates ... 1
2. Missing values ... 3
3. Unweighted characteristics of participants ... 7
4. Sensitivity analysis: adjusting for drinking/smoking status ... 9
5. Sensitivity analysis: Depression/anxiety ... 10
6. Complete case analysis ... 11
7. Multiple imputation assuming mental health data missing at random ... 13
8. People who smoked in the past year and drank at risky levels ... 15
9. Sensitivity analysis: adjusting for region across Great Britain ... 16

1. Covariates

Past-year smoking

Binary variable measured with the question *"Which of the following best applies to you?"*.

Answer options included:

i. *"I smoke cigarettes (including hand rolled) every day."*
ii. *"I smoke cigarettes (including hand rolled), but not every day."*
iii. *"I do not smoke cigarettes at all, but I do smoke tobacco of some kind (e.g. Pipe, Cigar or Shisha)."*
iv. *"I have stopped smoking completely in the last year."*
v. *"I stopped smoking completely more than a year ago."*
vi. *"I have never been a smoker (ie. smoked for a year or more)."*.

Those who replied with one of options (i) to (iv) were classified as having smoked in the past year.

Tobacco dependence was measured among those who smoked in the past year. The first category was 'not at all' (question: *"How much of the time have you felt the urge to smoke in the past 24 hours?"*). Other categories were 'Slight', 'Moderate', 'Strong', 'Very strong', and 'Extremely strong' (question: *"In general, how strong have the urges to smoke been?"*).

Alcohol Use Disorder Identification Test – Consumption (AUDIT-C)

The survey introduced these questions with the following:

"These first few questions ask about the alcohol you have drunk in the last 6 months, including about how many standard drinks you have consumed. Please note that 1 standard drink equals 1 unit of alcohol. So, for example, a small glass of wine or a single measure of spirits is 1 standard drink, while a pint of regular beer or lager is equal to 2 standard drinks or 2 units, and a bottle of wine is equal to 9 units. If you are unsure, please ask me to help you work it out.

Please be aware that all your answers will be handled confidentially."

AUDIT-C question 1: *"How often do you have a drink containing alcohol?*

i. *Never [value: 0]*
ii. *Monthly or less [value: 1]*
iii. *2 to 4 times a month [value: 2]*
iv. *2 to 3 times a week [value: 3]*
v. *4 to 5 times a week [value: 4]*
vi. *6 or more times a week [value: 4]*
vii. *Don't know*
viii. *Refused"*

The following questions are asked to all except those who answered (i), (vii), or (viii) to AUDIT-C question 1.

AUDIT-C question 2: *"How many standard drinks containing alcohol do you have on a typical day when you are drinking?*

i. *1 to 2 [value: 0]*
ii. *3 to 4 [value: 1]*
iii. *5 to 6 [value: 2]*
iv. *7 to 9 [value: 3]*
v. *10 to 12 [value: 4]*
vi. *13 to 15 [value: 4]*
vii. *16 or more [value: 4]*
viii. *Don't know*
ix. *Refused"*

AUDIT-C question 3: *"How often do you have six or more standard drinks on one occasion?*

i. *Never [value: 0]*
ii. *Less than monthly [value: 1]*
iii. *Monthly [value: 2]*
iv. *Weekly [value: 3]*
v. *Daily or almost daily [value: 4]*
vi. *Don't know*
vii. *Refused"*

Risky drinking – binary variable, measured using the AUDIT-C (i.e., AUDIT questions 1-3), with a score of 5 or above indicating risky drinking.

Alcohol consumption level – ordinal variable, ranging from 5 to 12 based on AUDIT-C, measured among those drinking at risky levels.

Survey wave ranged from 1 (October 2020) to 33 (June 2023).

Gender was categorised as woman, man, or non-binary. Due to small numbers, those categorised as non-binary were excluded from regression analyses.

Nation was classified as England, Scotland, or Wales.

Socioeconomic position was based on the National Readership Survey's classification of social grade:

- more advantaged social grades (ABC1: high and intermediate managerial, administrative, or professional, supervisory, clerical, and junior managerial, administrative or professional);
- less advantaged social grades (C2DE: skilled manual workers, semi and unskilled manual workers, state pensioners, casual or lowest grade workers, unemployed with state benefits only).

2. Missing values

Table S1: Missing values for each variable included in the analysis (N=23,790).

	**Missing values, n (%)**
Age	0 (0.0)
Gender	31 (0.1)
Social grade	0 (0.0)
Nation	0 (0.0)
History of a mental health condition	544 (2.3)
Smoking status	118 (0.5)
AUDIT-C[1] score	480 (2.0)
Tobacco dependence[2]	455 (4.6)
Smoking brief intervention/seeing GP[2]	253 (2.6)
Alcohol brief intervention/seeing GP[3]	200 (1.1)
Past-year smoking quit attempt[2]	508 (5.1)
Past-year alcohol reduction attempt[3]	0 (0)
Smoking quit attempt triggered by healthcare professional[2]	508 (5.1)
Alcohol reduction attempt triggered by healthcare professional[3]	10 (0.1)

[1] Alcohol use disorder identification test – consumption. [2] Among people who smoked in the past year.
[3] Among people drinking at risky levels.

We used the mice package in R, setting the function to 5 imputations and 5 iterations. For the variables ‘history of mental health condition’, ‘smoking brief intervention/seeing GP’, and ‘alcohol brief intervention/seeing GP’, values were imputed for each answer option separately. After the missing values were imputed, the variables used in the analyses were derived.

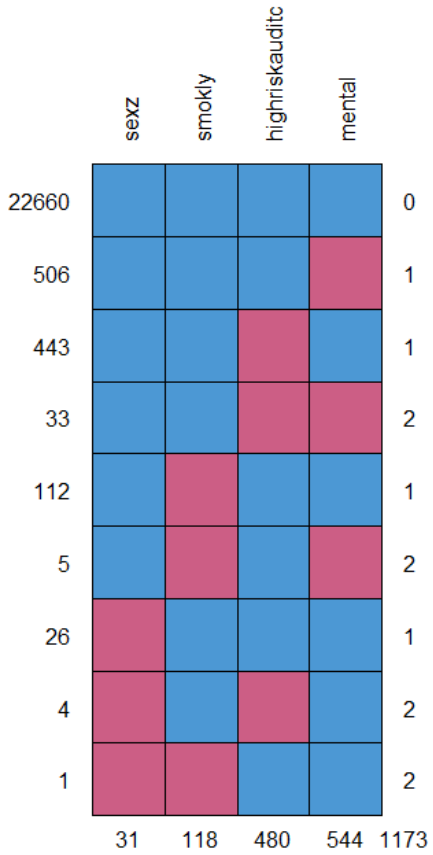

Figure S1: Patterns of missing data for all variables asked to all participants which had missing values (‘sexz’: gender; ‘smokly’: smoking status; ‘highriskauditc’: AUDIT-C score; ‘mental’: history of a mental health condition).

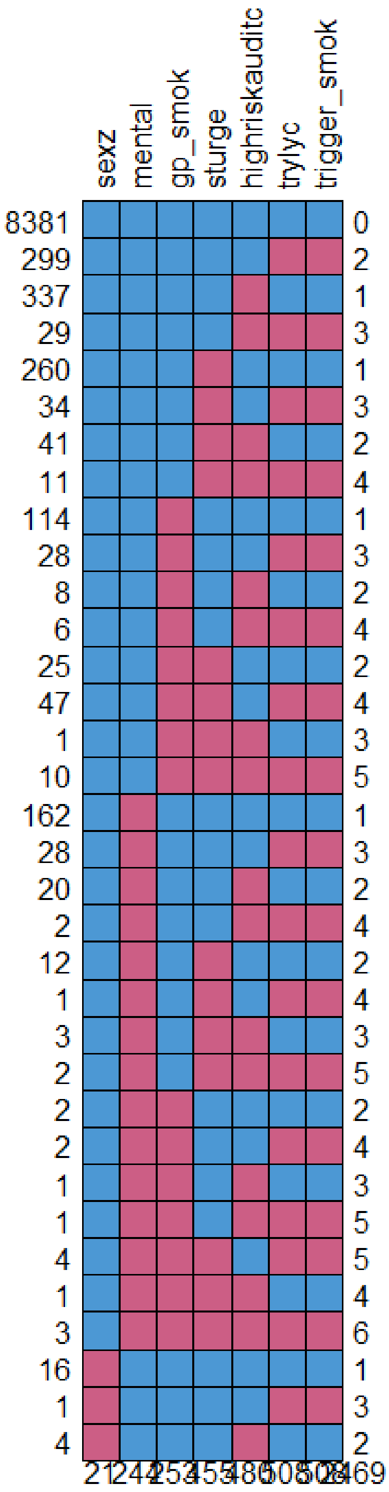

Figure S2: Patterns of missing data for all variables asked to people who smoked in the past year, which had missing values (‘sexz’: gender; ‘mental’: history of a mental health condition; ‘gp_smok’: smoking brief intervention/seeing GP; ‘sturge’: tobacco dependence; ‘highriskauditc’: AUDIT-C score; ‘trylyc’: past-year smoking quit attempt; ‘trigger_smok’: smoking quit attempt tiggered by healthcare professional). The numbers is the line below the graph should read as follows: 21, 244, 253, 455, 480. 508, 508, 2469.

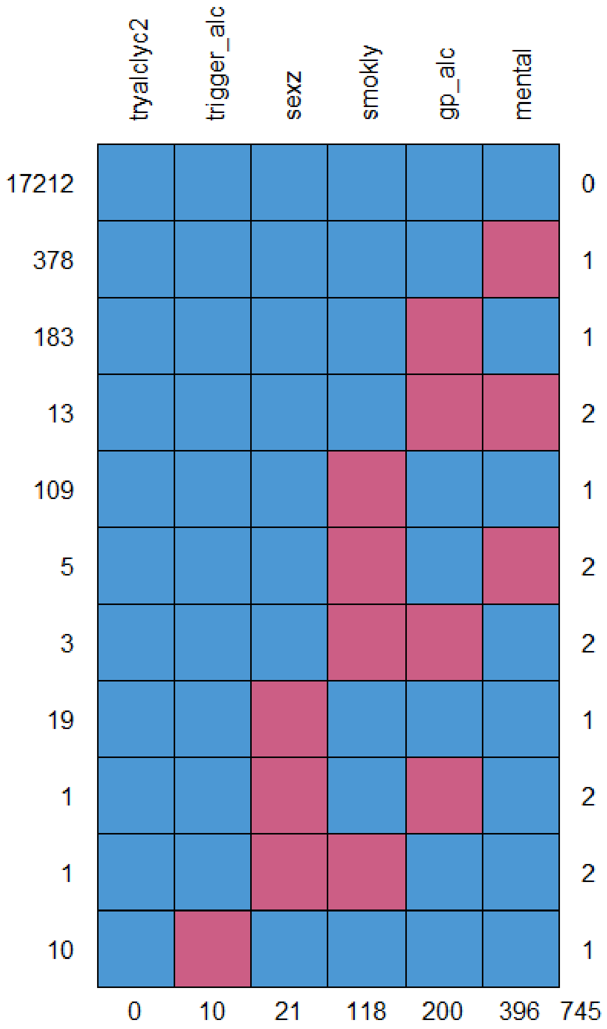

Figure S3: Patterns of missing data for all variables asked to people who drank at risky levels, which had missing values (‘trylalcyc2’: past-year alcohol reduction attempt; ‘trigger_alc’: alcohol reduction attempt tiggered by healthcare professional; ‘sexz’: gender; ‘smokly’: smoking status; ‘gp_alc’: alcohol brief intervention/seeing GP; ‘mental’: history of a mental health condition).

Table S2: Variables with missing values and how they were imputed.

Variable with missing values	Imputation method and conditions for imputation	Variables used for imputation
Gender	Polytomous logistic regression	Age, nation, social grade, history of mental health condition, AUDIT-C score, smoking status
History of mental health condition (all conditions separately)	Logistic regression with specified missing not at random condition using adjustment factor of 1.5, which assumes that, among people not answering (don't know or refused responses), the odds of having a history of the respective condition is 1.5-times higher than among people who responded to the question	Gender, age, nation, social grade history of mental health condition (all other conditions), AUDIT-C score, smoking status
Smoking status	Logistic regression	Gender, age, nation, social grade history of mental health condition, AUDIT-C score
AUDIT-C score	Predictive mean matching	Gender, age, nation, social grade history of mental health condition, smoking status
Tobacco dependence	Predictive mean matching; Only predicted if smoking	Gender, age, nation, social grade history of mental health condition, AUDIT-C score
Smoking brief intervention/ seeing GP	Logistic regression; Only predicted if smoking; only predict type of GP interaction if someone reported seeing GP; only predict type of recommendation if someone received brief intervention	Gender, age, nation, social grade history of mental health condition, AUDIT-C score, tobacco dependence
Alcohol brief intervention/ seeing GP	Logistic regression; Only predicted if AUDIT-C ≥5; only predict type of GP interaction if someone reported seeing GP only predict type of recommendation if someone received brief intervention	Gender, age, nation, social grade history of mental health condition, smoking status, AUDIT-C score
Past-year smoking quit attempt	Logistic regression; Only predicted if smoking	Gender, age, nation, social grade history of mental health condition, AUDIT-C score, tobacco dependence
Past-year alcohol reduction attempt	Logistic regression; Only predicted if AUDIT-C ≥5	Gender, age, nation, social grade history of mental health condition, smoking status, AUDIT-C score, tobacco dependence
Smoking quit attempt triggered by healthcare professional	Logistic regression; Only predicted if smoking and trying to quit	Gender, age, nation, social grade history of mental health condition, AUDIT-C score, tobacco dependence
Alcohol reduction attempt triggered by healthcare professional	Logistic regression; Only predicted if AUDIT-C ≥5 and trying to reduce consumption	Gender, age, nation, social grade history of mental health condition, smoking status, AUDIT-C score

3. Unweighted characteristics of participants

Table S3: Characteristics of participants ($N_{unweighted}$=23,790, data in table unweighted)

	**Only past-year smoking, % (95% CI)**		**Only risky drinking, % (95% CI)**		**Past-year smoking and risky drinking, % (95% CI)**	
	**History of mental health condition**					
	**Yes (n=2733)**	**No (n=2911)**	**Yes (n=3786)**	**No (n=10068)**	**Yes (n=1909)**	**No (n=2383)**
18-24 years	14.7 (13.4, 16.0)	10.0 (8.9, 11.1)	12.0 (11.0, 13.1)	8.4 (7.8, 8.9)	24.0 (22.0, 25.9)	16.5 (15.0, 18.0)
25-34 years	24.9 (23.3, 26.5)	18.2 (16.8, 19.6)	16.4 (15.2, 17.6)	12.3 (11.6,12.9)	26.9 (24.9, 28.9)	21.8 (20.2, 23.5)
35-44 years	17.6 (16.1, 19.0)	14.1 (12.9, 15.4)	17.1 (15.9, 18.3)	13.6 (12.9, 14.3)	17.9 (16.1, 19.6)	16.7 (15.2, 18.2)
45-54 years	16.8 (15.4, 18.2)	15.7 (14.4, 17.0)	20.8 (19.5, 22.1)	21.7 (20.9, 22.5)	16.5 (14.8, 18.2)	18.6 (17.1, 20.2)
55-64 years	14.2 (12.9, 15.6)	16.0 (14.7, 17.4)	18.8 (17.5, 20.0)	20.5 (19.8, 21.3)	9.7 (8.3, 11.0)	14.9 (13.5, 16.3)
65+ years	11.8 (10.6, 13.0)	26.0 (24.4, 27.5)	14.9 (13.8, 16.1)	23.6 (22.8, 24.4)	5.1 (4.1, 6.1)	11.5 (10.2, 12.7)
Women	59.1 (57.3, 61.0)	47.4 (45.6, 49.3)	51.1 (49.5, 52.7)	33.0 (32.1, 33.9)	47.8 (45.6, 50.1)	32.5 (30.6, 34.4)
Men	39.2 (37.4, 41.1)	52.2 (50.4, 54.0)	48.0 (46.4, 49.6)	66.7 (65.8, 67.6)	50.4 (48.2, 52.7)	67.4 (65.6, 69.3)
Non-binary	1.6 (1.2, 2.1)	0.4 (0.1, 0.6)	1.0 (0.7, 1.3)	0.3 (0.2, 0.4)	1.8 (1.2, 2.3)	0.1 (0.0, 0.2)
ABC1	45.5 (43.6, 47.3)	52.4 (50.6, 54.2)	71.3 (69.9, 72.7)	75.3 (74.4, 76.1)	58.4 (56.2, 60.7)	65.0 (63.1, 66.9)
C2DE	54.5 (52.7, 56.4)	47.6 (45.8, 49.4)	28.7 (27.3, 30.1)	24.7 (23.9, 25.6)	41.6 (39.3, 43.8)	35.0 (33.1, 36.9)
England	80.5 (79.0, 82.0)	80.5 (79.0, 81.9)	77.2 (75.9, 78.6)	77.3 (76.4, 78.1)	80.9 (79.1, 82.6)	80.5 (78.9, 82.1)
Scotland	12.4 (11.2, 13.6)	12.2 (11.0, 13.4)	15.5 (14.4, 16.7)	15.7 (15.0, 16.5)	12.3 (10.8, 13.8)	13.6 (12.2, 15.0)
Wales	7.1 (6.2, 8.1)	7.3 (6.4, 8.3)	7.2 (6.4, 8.0)	7.0 (6.5, 7.5)	6.9 (5.7, 8.0)	5.9 (5.0, 6.9)

Social grades ABC1: more socioeconomically advantaged; Social grades C2DE: less socioeconomically advantaged

Table S4: Unweighted results for GP visit, receipt of brief intervention, and quit attempts by history of mental health condition for people who smoked in the past year or who drank at risky levels.

		History of mental health condition			
		**Yes**, % (95% CI)	**No**, % (95% CI)	**OR** (95% CI), ref: no	$\mathbf{OR_{adj}}$[1] (95% CI), ref: no
Past-year smoking (n=9931)	GP visit	65.9 (64.6, 67.3)	50.7 (49.4, 52.1)	–	–
	SBI receipt	41.4 (39.7, 43.2)	41.2 (39.4, 43.1)	1.01 (0.91, 1.12)	1.04 (0.93, 1.17)
	Quit attempt	39.5 (38.0, 40.9)	33.6 (32.4, 34.9)	–	–
	Triggered by healthcare professional	11.0 (9.6, 12.4)	9.5 (8.1, 10.8)	1.18 (0.95, 1.47)	1.18 (0.94, 1.49)
Risky drinking (n=18149)	GP visit	69.8 (68.6, 71.0)	55.9 (55.0, 56.8)	–	–
	ABI receipt	6.6 (5.9, 7.4)	2.9 (2.5, 3.2)	2.42 (2.01, 2.93)	2.50 (2.04, 3.05)
	Cut-down attempt	40.8 (39.5, 42.1)	31.8 (31.0, 32.7)	–	–
	Triggered by healthcare professional	8.8 (7.6, 9.9)	5.5 (4.8, 6.3)	1.64 (1.35, 2.00)	1.87 (1.52, 2.30)
SBI (n=2384)	Advice only	41.4 (38.6, 44.1)	41.2 (38.3, 44.1)	1.00 (0.86, 1.19)	1.01 (0.84, 1.20)
	E-cigarette	10.9 (9.2, 12.6)	7.7 (6.1, 9.3)	1.46 (1.10, 1.95)	1.33 (0.99, 1.79)
	Prescription	24.3 (21.9, 26.6)	23.0 (20.5, 25.5)	1.07 (0.89, 1.30)	1.10 (0.90, 1.35)
	Referral to practice nurse	18.1 (16.0, 20.3)	19.1 (16.8, 21.5)	0.94 (0.76, 1.15)	1.00 (0.80, 1.25)
	Referral to SSS	38.7 (36.0, 41.4)	35.0 (32.2, 37.8)	1.17 (0.99, 1.38)	1.13 (0.95, 1.35)
ABI (n=462)	Advice only	70.0 (64.4, 75.5)	90.6 (86.6, 94.7)	0.24 (0.14, 0.41)	0.33 (0.17, 0.60)
	Support within the practice	31.2 (25.6, 36.8)	11.7 (7.2, 16.1)	3.43 (2.10, 5.79)	3.19 (1.79, 5.87)
	Referral to alcohol service/ specialist	34.1 (28.4, 39.9)	5.2 (2.1, 8.3)	9.40 (4.99, 19.59)	7.79 (4.03, 16.29)

[1] adjusted for age, gender, social grade, tobacco dependence or AUDIT-C score, nation, and survey wave.
Abbreviations: ABI, alcohol brief intervention; CI, confidence interval; GP, general practitioner; OR, odds ratio; OR_{adj}, adjusted odds ratio; SBI, smoking brief intervention; SSS, stop smoking service.

4. Sensitivity analysis: adjusting for drinking/smoking status

Table S5: Weighted results for GP visit, receipt of brief intervention, and quit attempts by history of mental health condition for people who smoked in the past year or who drank at risky levels.

		History of mental health condition
		**OR_{adj}**[1] (95% CI), ref: no
Past-year smoking (n=9936)	SBI receipt	1.01 (0.89, 1.15)
	Triggered by healthcare professional	1.17 (0.91, 1.52)
Risky drinking (n=18146)	ABI receipt	2.56 (2.06, 3.18)
	Triggered by healthcare professional	1.95 (1.57, 2.43)
SBI (n=2504)	Advice only	1.03 (0.85, 1.24)
	E-cigarette	1.28 (0.92, 1.80)
	Prescription	1.09 (0.87, 1.37)
	Referral to practice nurse	1.04 (0.82, 1.33)
	Referral to stop smoking service	1.05 (0.86, 1.27)
ABI (n=469)	Advice only	0.32 (0.17, 0.62)
	Support within the practice	3.58 (1.89, 6.79)
	Referral to alcohol service/specialist	8.51 (3.80, 19.10)

[1] adjusted for age, gender, social grade, risky drinking and tobacco dependence (if past-year smoking) or past-year smoking and AUDIT-C score (if risky drinking), nation, and survey wave. Abbreviations: ABI, alcohol brief intervention; CI, confidence interval; GP, general practitioner; OR_{adj}, adjusted odds ratio; SBI, smoking brief intervention.

5. Sensitivity analysis: Depression/anxiety

Table S6: Weighted results for GP visit, receipt of brief intervention, and quit attempts by history of depression or anxiety for people who smoked in the past year or who drank at risky levels.

		History of depression or anxiety			
		**Yes**, % (95% CI)	**No**[1], % (95% CI)	**OR** (95% CI), ref: no	**OR_{adj}**[2] (95% CI), ref: no
Past-year smoking (n=10021)	GP visit	66.1 (64.5, 67.7)	50.3 (48.8, 51.8)	–	–
	SBI receipt	41.6 (39.6, 43.6)	41.1 (39.0, 43.1)	1.02 (0.91, 1.15)	1.04 (0.91, 1.18)
	Quit attempt	40.3 (38.7, 42.0)	34.5 (33.1, 35.9)	–	–
	Triggered by healthcare professional	11.9 (10.2, 13.7)	9.6 (8.1, 11.1)	1.28 (1.01, 1.63)	1.27 (0.97, 1.65)
Risky drinking (n=4308)	GP visit	69.8 (68.4, 71.2)	55.1 (54.1, 56.0)	–	–
	ABI receipt	7.1 (6.1, 8.0)	2.8 (2.4, 3.2)	2.63 (2.13, 3.24)	2.76 (2.21, 3.43)
	Cut-down attempt	42.1 (40.6, 43.6)	31.6 (30.7, 32.4)	–	–
	Triggered by healthcare professional	9.4 (8.0, 10.7)	5.4 (4.7, 6.2)	1.81 (1.46, 2.25)	2.09 (1.67, 2.61)
SBI (n=883)	Advice only	41.4 (38.3, 44.6)	41.0 (37.8, 44.2)	1.02 (0.85, 1.22)	1.02 (0.84, 1.24)
	E-cigarette	10.8 (8.9, 12.7)	8.1 (6.3, 9.9)	1.37 (1.00, 1.88)	1.28 (0.90, 1.81)
	Prescription	24.3 (21.6, 27.0)	23.3 (20.6, 26.0)	1.06 (0.85, 1.31)	1.11 (0.88, 1.40)
	Referral to practice nurse	18.5 (16.0, 20.9)	18.8 (16.3, 21.3)	0.98 (0.78, 1.23)	1.04 (0.81, 1.33)
	Referral to SSS	38.1 (35.0, 41.1)	36.2 (33.1, 39.4)	1.08 (0.90, 1.31)	1.01 (0.83, 1.25)
ABI (n=441)	Advice only	69.2 (62.8, 75.6)	90.1 (85.4, 94.8)	0.25 (0.13, 0.46)	0.29 (0.15, 0.58)
	Support within the practice	31.3 (24.9, 37.6)	11.3 (6.3, 16.2)	3.58 (2.02, 6.38)	3.81 (1.95, 7.44)
	Referral to alcohol service/ specialist	35.7 (29.0, 42.4)	6.0 (2.1, 9.9)	8.73 (4.14, 18.40)	9.42 (4.08, 21.76)

[1] 'No' means no history of mental health condition. [2] Adjusted for age, gender, social grade, tobacco dependence or AUDIT-C score, nation, and survey wave. Abbreviations: ABI, alcohol brief intervention; CI, confidence interval; GP, general practitioner; OR, odds ratio; OR_{adj}, adjusted odds ratio; SBI, smoking brief intervention; SSS, stop smoking service.

6. **Complete case analysis**

Table S7: Characteristics of participants ($N_{unweighted}$=21,718, data in table weighted).

	Only past-year smoking, % (95% CI)		Only risky drinking, % (95% CI)		Past-year smoking and risky drinking, % (95% CI)	
	History of mental health condition					
	Yes (n=2241)	No (n=2462)	Yes (n=3550)	No (n=9829)	Yes (n=1606)	No (n=2030)
18-24 years	15.1 (13.5, 16.7)	11.2 (9.8, 12.7)	13.1 (11.9, 14.3)	9.3 (8.6, 10.0)	25.8 (23.4, 28.2)	17.5 (15.6, 19.3)
25-34 years	29.3 (27.2, 31.4)	21.2 (19.3, 23.1)	18.9 (17.4, 20.3)	14.3 (13.5, 15.1)	27.9 (25.4, 30.3)	25.3 (23.1, 27.4)
35-44 years	19.1 (17.3, 20.9)	16.0 (14.3, 17.6)	18.7 (17.3, 20.2)	15.9 (15.1, 16.7)	19.1 (16.9, 21.2)	18.7 (16.8, 20.6)
45-54 years	15.3 (13.7, 16.9)	14.9 (13.5, 16.4)	19.4 (18.0, 20.8)	20.8 (19.9, 21.6)	15.5 (13.6, 17.3)	17.3 (15.6, 19.1)
55-64 years	12.0 (10.6, 13.3)	14.6 (13.2, 16.1)	17.1 (15.8, 18.4)	19.1 (18.3, 19.9)	7.6 (6.3, 8.9)	12.9 (11.4, 14.5)
65+ years	9.2 (8.0, 10.4)	22.0 (20.3, 23.7)	12.8 (11.7, 14.0)	20.7 (19.8, 21.5)	4.2 (3.2, 5.2)	8.3 (7.1, 9.4)
Women	58.5 (56.3, 60.8)	46.0 (43.8, 48.1)	52.5 (50.4, 53.9)	33.0 (32.0, 34.0)	47.2 (44.6, 49.9)	32.5 (30.3, 34.7)
Men	39.9 (37.7, 42.2)	53.8 (51.6, 55.9)	46.9 (45.1, 48.7)	66.6 (65.6, 67.6)	50.2 (47.5, 52.9)	67.1 (64.9, 69.3)
Non-binary	1.5 (1.0, 2.0)	0.3 (0.1, 0.5)	1.0 (0.6, 1.3)	0.3 (0.2, 0.5)	2.6 (1.8, 3.3)	0.4 (0.1, 0.7)
ABC1	35.2 (33.2, 37.2)	40.9 (38.9, 43.0)	61.8 (60.0, 63.6)	66.6 (65.5, 67.7)	47.0 (44.4, 49.7)	53.2 (50.7, 55.6)
C2DE	64.8 (62.8, 66.8)	59.1 (57.0, 61.1)	38.2 (36.4, 40.0)	33.4 (32.3, 34.5)	53.0 (50.3, 55.6)	46.8 (44.4, 49.3)
England	84.8 (83.4, 86.3)	85.2 (83.8, 86.6)	82.2 (81.0, 83.5)	82.4 (81.6, 83.1)	85.3 (83.6, 87.0)	84.7 (83.2, 86.3)
Scotland	9.4 (8.2, 10.5)	8.9 (7.8, 10.0)	12.0 (10.9, 13.0)	12.1 (11.5, 12.7)	9.2 (7.9, 10.6)	10.4 (9.1, 11.6)
Wales	5.8 (4.8, 6.8)	5.9 (5.0, 6.8)	5.8 (5.0, 6.6)	5.5 (5.1, 6.0)	5.5 (4.3, 6.6)	4.9 (4.0, 5.9)

Social grades ABC1: more socioeconomically advantaged; Social grades C2DE: less socioeconomically advantaged.

Table S8: GP visit and receipt of brief intervention by history of mental health condition for people who smoked in the past year or who drank at risky levels (complete cases, $N_{unweighted}$=21,718, data in table weighted).

		**History of mental health condition**			
		Yes, % (95% CI)	No, % (95% CI)	OR (95% CI), ref: no	OR_{adj}[1] (95% CI), ref: no
Past-year smoking (n=8915)	GP visit	65.6 (63.9, 67.2)	50.1 (48.5, 51.7)	–	–
	SBI receipt	42.1 (40.0, 44.1)	42.5 (40.3, 44.7)	0.98 (0.87, 1.11)	1.01 (0.88, 1.16)
	Quit attempt	40.9 (39.2, 42.6)	35.3 (33.8, 36.9)	–	–
	Triggered by healthcare professional	10.7 (9.0, 12.4)	9.0 (7.4, 10.5)	1.22 (0.93, 1.58)	1.21 (0.90, 1.61)
Risky drinking (n=16719)	GP visit	70.5 (69.2, 71.9)	54.8 (53.8, 55.8)	–	–
	ABI receipt	7.4 (6.4, 8.3)	2.6 (2.2, 3.0)	3.01 (2.43, 3.73)	3.15 (2.51, 3.94)
	Cut-down attempt	41.8 (40.3, 43.3)	31.6 (30.7, 32.5)	–	–
	Triggered by healthcare professional	8.6 (7.3, 9.9)	4.5 (3.8, 5.2)	2.00 (1.59, 2.53)	2.31 (1.83, 2.93)
SBI (n=2160)	Advice only	42.1 (39.0, 45.3)	41.3 (37.9, 44.8)	1.03 (0.85, 1.25)	1.04 (0.85, 1.28)
	E-cigarette	11.2 (9.1, 13.2)	8.2 (6.3, 10.1)	1.40 (1.02, 1.94)	1.34 (0.94, 1.91)
	Prescription	24.1 (21.4, 26.8)	22.6 (19.7, 25.4)	1.09 (0.87, 1.36)	1.18 (0.93, 1.51)
	Referral to practice nurse	19.5 (16.9, 22.1)	19.1 (16.4, 21.7)	1.03 (0.81, 1.31)	1.09 (0.85, 1.41)
	Referral to SSS	38.9 (35.7, 42.0)	35.7 (32.4, 39.1)	1.14 (0.94, 1.39)	1.08 (0.88, 1.34)
ABI (n=434)	Advice only	67.5 (61.2, 73.8)	92.8 (88.7, 96.9)	0.16 (0.08, 0.32)	0.19 (0.09, 0.40)
	Support within the practice	31.8 (25.6, 38.0)	10.0 (5.1, 14.9)	4.21 (2.27, 7.78)	4.36 (2.14, 8.91)
	Referral to alcohol service/ specialist	34.9 (28.4, 41.3)	4.0 (0.5, 7.5)	12.80 (4.93, 33.20)	11.70 (4.47, 30.80)

[1] adjusted for age, gender, social grade, tobacco dependence or AUDIT-C score, nation, and survey wave.
Abbreviations: ABI, alcohol brief intervention; CI, confidence interval; GP, general practitioner; OR, odds ratio; OR_{adj}, adjusted odds ratio; SBI, smoking brief intervention; SSS, stop smoking service.

7. Multiple imputation assuming mental health data missing at random

Table S9: Characteristics of participants (imputed, missing at random, $N_{unweighted}$=27,937, data in table weighted).

	Only past-year smoking, % (95% CI)		Only risky drinking, % (95% CI)		Past-year smoking and risky drinking, % (95% CI)	
	History of mental health condition					
	Yes (n=2875)	No (n=3167)	Yes (n=3587)	No (n=9729)	Yes (n=2020)	No (n=2547)
18-24 years	15.5 (14.0, 17.0)	11.5 (10.2, 12.9)	13.1 (11.9, 14.3)	9.2 (8.6, 9.9)	25.8 (23.6, 28.0)	17.0 (15.3, 18.7)
25-34 years	28.0 (26.0, 29.9)	21.6 (19.9, 23.4)	18.9 (17.4, 20.4)	14.3 (13.6, 15.1)	28.7 (26.4, 30.9)	25.1 (23.1, 27.1)
35-44 years	19.0 (17.3, 20.7)	15.8 (14.3, 17.3)	19.0 (17.6, 20.5)	15.9 (15.1, 16.7)	19.0 (17.0, 21.0)	18.5 (16.7, 20.2)
45-54 years	15.7 (14.2, 17.2)	14.9 (13.5, 16.2)	19.5 (18.2, 20.9)	20.8 (19.9, 21.6)	14.6 (12.9, 16.3)	17.1 (15.5, 18.7)
55-64 years	12.1 (10.8, 13.3)	14.0 (12.7, 15.3)	16.9 (15.6, 18.1)	19.0 (18.2, 19.8)	8.0 (6.7, 9.3)	13.3 (11.9, 14.7)
65+ years	9.8 (8.6, 10.9)	22.2 (20.6, 23.7)	12.6 (11.5, 13.7)	20.8 (20.0, 21.6)	4.0 (3.1, 4.9)	9.1 (8.0, 10.2)
Women	58.3 (56.3, 60.4)	45.0 (43.1, 47.0)	52.1 (50.3, 53.8)	32.8 (31.9, 33.8)	46.6 (44.1, 49.0)	31.3 (29.3, 33.3)
Men	40.1 (38.1, 42.2)	54.6 (52.7, 56.6)	47.0 (45.2, 48.7)	66.8 (65.8, 67.8)	50.6 (48.1, 53.0)	68.1 (66.1, 70.1)
Non-binary	1.5 (1.1, 2.0)	0.3 (0.1, 0.6)	1.0 (0.6, 1.3)	0.4 (0.2, 0.5)	2.9 (2.1, 3.6)	0.5 (0.3, 0.8)
ABC1	34.8 (33.0, 36.7)	41.1 (39.3, 43.0)	61.7 (59.9, 63.5)	66.4 (65.4, 67.5)	47.5 (45.1, 50.0)	52.6 (50.3, 54.8)
C2DE	65.2 (63.3, 67.0)	58.9 (57.0, 60.7)	38.3 (36.5, 40.1)	33.6 (32.5, 34.6)	52.5 (50.0, 54.9)	47.4 (45.2, 49.7)
England	85.1 (83.8, 86.4)	85.0 (83.8, 86.3)	82.3 (81.0, 83.5)	82.4 (81.7, 83.1)	85.1 (83.5, 86.7)	85.2 (83.8, 86.6)
Scotland	9.1 (8.1, 10.2)	9.0 (8.1, 10.0)	12.0 (11.0, 13.0)	12.0 (11.4, 12.7)	9.2 (7.9, 10.5)	9.9 (8.8, 11.1)
Wales	5.7 (4.9, 6.6)	5.9 (5.1, 6.8)	5.8 (5.0, 6.5)	5.5 (5.1, 6.0)	5.7 (4.6, 6.7)	4.8 (4.0, 5.7)

Social grades ABC1: more socioeconomically advantaged; Social grades C2DE: less socioeconomically advantaged.

Table S10: GP visit and receipt of brief intervention by history of mental health condition for people who smoked in the past year or who drink at risky levels (imputed, missing at random, $N_{unweighted}$=27,937, data weighted).

		History of mental health condition			
		Yes, % (95% CI)	No, % (95% CI)	OR (95% CI), ref: no	OR_{adj}[1] (95% CI), ref: no
Past-year smoking (n=9936)	GP visit	65.5 (64.0, 67.0)	50.4 (49.0, 51.9)	–	–
	SBI receipt	41.2 (39.3, 43.1)	41.1 (39.1, 43.1)	1.00 (0.90, 1.13)	1.02 (0.90, 1.16)
	Quit attempt	40.2 (38.7, 41.8)	34.6 (33.2, 36.0)	–	–
	Triggered by healthcare professional	11.3 (9.7, 12.9)	9.6 (8.1, 11.1)	1.20 (0.95, 1.52)	1.19 (0.92, 1.54)
Risky drinking (n=18145)	GP visit	70.1 (68.8, 71.4)	55.1 (54.1, 56.0)	–	–
	ABI receipt	7.0 (6.1, 7.9)	2.9 (2.4, 3.3)	2.57 (2.10, 3.16)	2.70 (2.18, 3.35)
	Cut-down attempt	41.2 (39.8, 42.6)	31.6 (30.8, 32.5)	–	–
	Triggered by healthcare professional	8.8 (7.5, 10.1)	5.5 (4.8, 6.3)	1.66 (1.34, 2.06)	1.94 (1.55, 2.41)
SBI (n=2505)	Advice only	41.4 (38.4, 44.4)	41.0 (37.8, 44.1)	1.02 (0.85, 1.22)	1.03 (0.85, 1.24)
	E-cigarette	11.2 (9.3, 13.1)	7.9 (6.2, 9.7)	1.47 (1.08, 2.00)	1.38 (0.98, 1.93)
	Prescription	24.4 (21.8, 26.9)	23.3 (20.6, 26.0)	1.06 (0.86, 1.30)	1.11 (0.89, 1.40)
	Referral to practice nurse	18.7 (16.4, 21.1)	18.5 (16.1, 21.0)	1.01 (0.81, 1.27)	1.08 (0.85, 1.37)
	Referral to SSS	38.5 (35.6, 41.4)	36.2 (33.1, 39.3)	1.10 (0.92, 1.32)	1.04 (0.86, 1.26)
ABI (n=470)	Advice only	68.4 (62.2, 74.6)	90.2 (85.6, 94.8)	0.24 (0.13, 0.43)	0.30 (0.16, 0.57)
	Support within the practice	32.5 (26.3, 38.7)	10.9 (6.1, 15.7)	3.94 (2.23, 6.94)	4.01 (2.11, 7.65)
	Referral to alcohol service/ specialist	36.1 (29.7, 42.6)	6.5 (2.6, 10.4)	8.25 (4.09, 16.66)	8.22 (3.80, 17.80)

[1] adjusted for age, gender, social grade, tobacco dependence or AUDIT-C score, nation, and survey wave.
Abbreviations: ABI, alcohol brief intervention; CI, confidence interval; GP, general practitioner; OR, odds ratio; OR_{adj}, adjusted odds ratio; SBI, smoking brief intervention; SSS, stop smoking service.

8. **People who smoked in the past year and drank at risky levels**

Table S11: GP visit and receipt of brief intervention by history of mental health condition for people who smoked in the past year and drank at risky levels ($n_{weighted}$=4,577, data weighted).

	**History of mental health condition**			
	Yes, % (95% CI)	No, % (95% CI)	OR (95% CI), ref: no	OR_{adj}[1] (95% CI), ref: no
GP visit	73.5 (71.3, 75.6)	57.2 (55.1, 59.4)	–	–
SBI receipt	32.4 (29.7, 35.0)	31.5 (28.8, 34.2)	1.04 (0.87, 1.24)	1.02 (0.84, 1.23)
ABI receipt	9.1 (7.5, 10.8)	3.4 (2.3, 4.4)	2.88 (1.98, 4.18)	2.97 (2.01, 4.38)

[1] adjusted for age, gender, social grade, tobacco dependence or AUDIT-C score, nation, and survey wave.
Abbreviations: ABI, alcohol brief intervention; CI, confidence interval; GP, general practitioner; OR, odds ratio; OR_{adj}, adjusted odds ratio; SBI, smoking brief intervention; SSS, stop smoking service.

9. Sensitivity analysis: adjusting for region across Great Britain

Table S12: Weighted prevalence of history of a mental health condition by region across Great Britain.

	**History of mental health condition**, % (95% CI)
North East	37.3 (34.1, 40.6)
North West	38.8 (36.8, 40.9)
Yorkshire and The Humber	37.3 (34.9, 39.7)
East Midlands	38.2 (35.6, 40.9)
West Midlands	37.8 (33.0, 37.8)
East of England	38.4 (36.0, 40.8)
London	33.9 (32.0, 35.8)
South East	36.7 (34.8, 38.5)
South West	35.9 (33.6, 38.3)
Wales	37.7 (35.1, 40.3)
Scotland	35.0 (33.2, 36.7)

Table S13: Weighted results for GP visit, receipt of brief intervention, and quit attempts by history of mental health condition for people who smoked in the past year or who drank at risky levels.

		**History of mental health condition** $\mathbf{OR_{adj}}$[1] (95% CI), ref: no
Past-year smoking (n=9936)	SBI receipt	1.02 (0.90, 1.15)
	Triggered by healthcare professional	1.19 (0.92, 1.54)
Risky drinking (n=18146)	ABI receipt	2.70 (2.18, 3.35)
	Triggered by healthcare professional	2.01 (1.61, 2.49)
SBI (n=2504)	Advice only	1.01 (0.84, 1.23)
	E-cigarette	1.28 (0.91, 1.79)
	Prescription	1.10 (0.88, 1.38)
	Referral to practice nurse	1.03 (0.81, 1.31)
	Referral to stop smoking service	1.03 (0.85, 1.25)
ABI (n=469)	Advice only	0.31 (0.16, 0.60)
	Support within the practice	3.66 (1.93, 6.94)
	Referral to alcohol service/specialist	9.81 (4.35, 22.12)

[1] adjusted for age, gender, social grade, tobacco dependence (if past-year smoking) or AUDIT-C score (if risky drinking), region across Great Britain, and survey wave. Abbreviations: ABI, alcohol brief intervention; CI, confidence interval; GP, general practitioner; OR_{adj}, adjusted odds ratio; SBI, smoking brief intervention.
